# Effect of silicon nanoparticle-based biochar on wheat growth, antioxidants and nutrients concentration under salinity stress

**DOI:** 10.1038/s41598-024-55924-7

**Published:** 2024-03-16

**Authors:** Sidra Gill, Musarrat Ramzan, Gul Naz, Liaqat Ali, Subhan Danish, Mohammad Javed Ansari, Saleh H. Salmen

**Affiliations:** 1 Department of Botany, Faculty of Chemical and Biological Sciences, The Islamia University of Bahawalpur, Bahawalpur, Pakistan; 2 Cholistan Institute of Desert Studies, The Islamia University of Bahawalpur, Bahawalpur, Pakistan; 3 Department of Soil Science, Faculty of Agricultural Sciences and Technology, Bahauddin Zakariya University, Multan, Punjab, Pakistan; 4 Department of Botany, Hindu College Moradabad (MJP Rohilkhand University Bareilly), Moradabad, India 244001; 5 Department of Botany and Microbiology, College of Science, King Saud University, PO Box 2455, 11451 Riyadh, Saudi Arabia; 6 Institute of Physics, Faculty of Physics and Mathematics, The Islamia University of Bahawalpur, Bahawalpur, Pakistan

**Keywords:** Antioxidants, Biochar, Macronutrients, Micronutrients, Salinity stress, Silicon nanoparticles, Wheat, Plant stress responses, Abiotic, Salt

## Abstract

Globally, salinity is an important abiotic stress in agriculture. It induced oxidative stress and nutritional imbalance in plants, resulting in poor crop productivity. Applying silicon (Si) can improve the uptake of macronutrients. On the other hand, using biochar as a soil amendment can also decrease salinity stress due to its high porosity, cation exchange capacity, and water-holding capacity. That’s why the current experiment was conducted with novelty to explore the impact of silicon nanoparticle-based biochar (Si-BC) on wheat cultivated on salt-affected soil. There were 3 levels of Si-BC, i.e., control (0), 1% Si-BC1, and 2.5% Si-BC2 applied in 3 replicates under 0 and 200 mM NaCl following a completely randomized design. Results showed that treatment 2.5% Si-BC2 performed significantly better for the enhancement in shoot and root length, shoot and root fresh weight, shoot and root dry weight, number of leaves, number of tillers, number of spikelets, spike length, spike fresh and dry weight compared to control under no stress and salinity stress (200 mM NaCl). A significant enhancement in chlorophyll a (~ 18%), chlorophyll b (~ 22%), total chlorophyll (~ 20%), carotenoid (~ 60%), relative water contents (~ 58%) also signified the effectiveness of treatment 2.5% Si-BC2 than control under 200 mM NaCl. In conclusion, treatment 2.5% Si-BC2 can potentially mitigate the salinity stress in wheat by regulating antioxidants and improving N, K concentration, and gas exchange attributes while decreasing Na and Cl concentration and electrolyte leakage. More investigations at the field level are recommended for the declaration of treatment 2.5% Si-BC2 as the best amendment for alleviating salinity stress in different crops under variable climatic conditions.

## Introduction

Soil salinity is a major problem in agriculture, which significantly decreases crop productivity. It primarily harms plants through inhibition of leaf expansion, limited photosynthesis, decrease in chlorophyll contents, plant height, leaf area index, seed germination, uptake of water, and imbalance in nutrient uptake^1–3^. Furthermore, higher uptake of Na^+^ and Cl^−^ from the rhizosphere induced oxidative stress due to the generation of reactive oxygen species (ROS)^4,5^. Reactive oxygen species (ROS) can interact with vital components within plant cells, inducing oxidative harm. This oxidative damage can encompass lipid peroxidation, DNA impairment, protein oxidation, enzyme deactivation, and hormone disruption^6^.

To overcome this critical issue, the use of biochar is becoming popular. It is a porous carbon-rich organic amendment prepared by pyrolysis of biomass at high temperatures under limited or no supply of oxygen^7^. Due to its high aromaticity, biochar is very resistant and has a long carbon sequestering capacity^8^. It can potentially improve soil water holding capacity, permeability, and aeration^9^, soil organic matter content, soil nutrients content^10^, and crop yield^11^. In addition to the above, the application of biochar to salt-affected soils can benefit soil microbial activity. Improving soil microbial activities enhances the soil aggregate stability and releases nutrients for microbial utilization^12^. This stimulates root exudation of dissolved organic carbon and nitrogen, which are essential components in microbial metabolism^12^.

Silicon (Si) also likely plays a positive role in promoting plant growth when cultivated under salinity stress^2^. It has improved several aspects of plant physiology, including photosynthesis, redox balance, and nutrient management^13^. Additionally, it promotes root growth and helps maintain cell wall integrity, which is crucial for supporting selective permeability in plants^14^. This multifaceted role of silicon underscores its importance as a beneficial element for plant growth and development^13,14^. Applying silicon to plants can also significantly impact water regulation and hormonal balance. It aids in maintaining optimal water content within plants by enhancing water uptake efficiency and reducing water loss through transpiration^15^.

That’s why the current study explored the effects of silicon nanoparticles (SiNP) on wheat cultivated in salt-affected soils. The novelty of the current study lies in the use of SiNP-based biochar for the alleviation of salinity stress in wheat. The study aimed to improve wheat growth under salinity stress. This study covers the knowledge gap regarding the use of SiNP-based biochar for the mitigation of adverse effects on salinity. It is hypothesized that applying SiNP-based biochar might have the potential to minimize the adverse effects of salinity stress to improve wheat growth.

## Materials and methods

### Experimental site and soil collection

A pot experiment was conducted at the Islamia University of Bahawalpur, Botany Department, during the 2021–2022 wheat growing season. The soil was collected from the departmental nursery for the cultivation of wheat. The pre-experimental soil characteristics include pH*s* (8.45), EC*e* (3.24 dS/m), nitrogen (0.0025%), phosphorus (7.17 µg/g), exchangeable K (85 µg/g), and soil organic matter (0.35%).

### Synthesis of Silicon nanoparticle-based Biochar

Sugarcane press mud biochar synthesis was reported in a previous study. Biochar was produced using the technique described by^16^. Sugarcane press mud was air-dried before being pyrolyzed for 4 h at 450 °C in a muffle furnace^17^. Using the technique of^18^, the produced biochar was sampled, crushed, and sieved with a 0.250 mm strainer. Since the BC sample was collected from our nearby sugar industry (Ashraf Sugar Mill Ltd. BWP) and sugarcane-grown lands in our environment are silicon enriched, the traces of SiO_2_ dominate over other nutrients, i.e., P, Mg, Ca, etc.

### Characterization of Si-BC

Characterization of biochar was performed as previously described^19^. However, the surface morphology of sieved biochar (sBC) was observed using a ZEISS SEM microscope get on with a 15 kV accelerating voltage. To perform quantitative/elemental analysis, an energy dispersive X-ray (EDX) equipped with the SEM was employed on the sBC powder sample. This sample's X-ray diffraction (XRD) was carried out through Bruker-D8 Advance X-ray Diffractometer with Cu-*Kα* radiation (λ = 1.54 Å), set to 35 mA current with 40 kV applied potential. To analyze the structure, the XRD instrument scanned the sBC sample at room temperature in 20°–80°. To examine various functional groups present in sBC powder, a Tensor: 27 (Bruker) FTIR spectrometer was run in the frequency range 400–4000 cm^−1^, including a few mg of the powder sample mixed with KBr chemical to form a pellet for the analysis.

### Seeds collection

For experimental purposes, seeds of *Triticum aestivum* cultivar (ASS-2011) were purchased from a certified seed dealer in Bahawalpur. The seeds were initially screened out manually. After that, the seeds were sterilized using a 0.1% mercuric chloride solution for 5 min. Afterward, the seeds were rinsed with sterilized water 3 times to eliminate the residual effects of mercuric chloride.

### Pots preparation and seeds sowing

Plastic pots were used to conduct the experiment. The dimensions of pots were 20 cm in diameter and 30 cm deep. Each pot was filled with 6 kg of soil. A total of 15 seeds were sown initially in each pot. When seeds were germinated, thinning was performed to maintain 5 seedlings per pot.

### Fertilizer

N, P, and K were applied at the rate of 52, 46, and 25 kg/acre (0.39, 0.34, and 0.19 g/pot) for providing macronutrients. For nitrogen, urea fertilizer was used. However, for P and K, single superphosphate and potassium sulfate were used. Urea was applied in 3 splits, while P and K were applied in a single split at the time of pot preparation.

### Irrigation

The moisture contents of each pot were maintained at 65% field capacity regularly by using the moisture meter (YIERYI 4 in 1; Shenzhen, Guangdong Province, China).

### Treatment plan and experimental design

Before planting seedlings, SiNP-based biochar was mixed into the soil at 1 and 2.5% (w/w). The treatments include control (no Si-BC and NaCl), salinity stress (200 mM NaCl solution for irrigation), 1% SiNP-based biochar, 1% SiNP-based biochar + salinity stress, 2.5% SiNP-based biochar and 2.5% SiNP-based biochar + salinity stress (Figure S1). All the treatments were applied in 3 replicates following a completely randomized design (CRD).

### Harvesting and data collection

Plants were harvested after 75 days of sowing. The data regarding morphological attributes was collected soon after harvesting (Figure S2). For the fresh weight of samples, analytical balance was used. However, sample drying was done in an oven at 65 °C for 24 h to collect the dry weight data of samples.

### Chlorophyll estimation

For analysis of chlorophyll contents in the fresh leaves of wheat, samples were ground in 80% acetone. After that, filtration and absorbance were taken at 663 and 645 nm wavelengths on UV spectrophotometer^20^. The final values for chlorophyll a, b, and total were computed using the eq.

$$\begin{aligned} & {\text{Chlorophyll }}\;{\text{a }}\left( {\frac{{{\text{mg}}}}{{\text{g}}}} \right) = \frac{{\left( {12.7{ } \times {\text{ A}}663} \right){ }{-}{ }\left( {2.69{ } \times {\text{ A}}645} \right) \times {\text{V}}}}{{1000{ } \times {\text{W}}}} \\ & {\text{Chlorophyll }}\;{\text{b }}\left( {\frac{{{\text{mg}}}}{{\text{g}}}} \right) = \frac{{\left( {22.9{ } \times {\text{ A}}645} \right){ }{-}{ }\left( {4.68{ } \times {\text{ A}}663} \right) \times {\text{V}}}}{{1000{ } \times {\text{W}}}} \\ & {\text{Total}}\;{\text{Chlorophyll }}\left( {\frac{{{\text{mg}}}}{{\text{g}}}} \right) = \frac{{20.2\left( {{\text{A}}645} \right) + 8.02\left( {{\text{A}}663} \right) \times {\text{V}}}}{{1000{ } \times {\text{W}}}} \\ & {\text{Carotenoids }}\left( {\frac{{{\text{mg}}}}{{\text{g}}}} \right) = {\text{OD}}480 + 0.114{ }\left( {{\text{OD}}663} \right)-0.638{ }\left( {{\text{OD}}645} \right) \\ \end{aligned}$$

The photosyn Q meter version 2.0 was used to determine the characteristics of chlorophyll fluorescence.

### Leaf relative water content

Initially, 0.5 g of fresh-weight leaf sample was selected for analysis. The sample's turgid weight (TW) was then measured after immersing it in 100 ml of distilled water for 4 h, and the weight was recorded accordingly. The sample underwent oven drying at 70 °C for 48 h to obtain its dry weight (DW)^21^. The final values were obtained using the eq.

$${\text{RWC }}\left( \%  \right){ } = { }\left( {{\text{FW }} - {\text{ DW}}} \right){ }/{ }\left( {{\text{TW }} - {\text{ DW}}} \right){ } \times { }100$$

### Antioxidant enzyme

Nitro blue tetrazolium was used as per standard protocol for assessing the SOD activity by taking absorbance at 560 nm^22^. The enzymatic breakdown of hydrogen peroxide (H_2_O_2_) for CAT activity was determined by taking absorbance at 240 nm^23^. For APX activity, the reaction between ascorbic acid and H_2_O_2_ was noted at 290 nm wavelength^24^. However, malondialdehyde (MDA) was quantified via thiobarbituric acid method^25^.

### Electrolyte leakage

Leaf sections weighing one gram each were placed into individual test tubes containing 20 ml of deionized water. These test tubes were then maintained at a steady temperature of 25 °C for 24 h, after which the electrical conductivity of the solution (EC1) was assessed using a calibrated EC meter. Subsequently, the test tubes were heated at 120 °C for 20 min in a water bath, followed by the recording of the second electrical conductivity measurement (EC2)^26^.

$$ {\text{Electrolyte}}\;{\text{Leakage}}\;{ }\left( \% \right) = \left( {\frac{{{\text{EC}}1}}{{{\text{EC}}2}}} \right) \times { }100 $$

### Total soluble sugar and total soluble protein estimation

The soluble protein concentration was evaluated using the Bradford assay^27^. Fresh roots and shoots weighing 0.5 g each were homogenized in 10 mL of phosphate buffer with a pH of 7.8 and then centrifuged for 20 min at 10,000 revolutions per minute (rpm). Following centrifugation, 0.1 mL of the protein extraction was mixed with 0.9 mL of tris–HCl buffer and 5 mL of G-250 Coomassie reagent, and the mixture was left at room temperature for 2 min. Absorbance was measured at 595 nm using distilled water as the blank. The protein concentrations were determined using a bovine serum albumin (BSA) standard curve. 0.1 mL of plant extract was produced in 25 mL test tubes and used to estimate the soluble sugars using the^28^ method. Each tube was heated for 10 min in a boiling bath and then filled with 6 mL of anthrone reagent. After filling, the contents were solidified at room temperature for 10 min. The tubes were then incubated for an additional 20 min following solidification. Subsequently, the optical spectrum was read at 625 nm using a spectrophotometer.

### Ions estimation

To analyze Mn, Fe, Cu, Zn, K, and Na, samples were digested using a diacid mixture (nitric and perchloric acid in 3:1 ratio)^29^. The digested sample was run on a flame photometer to determine K and Na^30^. However, an atomic absorption spectrophotometer was used to compute Mn, Fe, Cu, and Zn. In the case of NO_3_, determination, sulfuric acid digestion was done at 450 °C by incorporating the digestion mixture of (CuSO_4_, FeSO_4_, and K_2_SO_4_)^31^.

### Statistical analysis

Standard statistical procedure was followed for the statistical analysis of the data^32^. Two factorial ANOVA was applied for the determination of significance. Each treatment was compared using the Tukey Test at *p* ≤ 0.05 using OriginPro software^33^. A paired comparison was applied to make the graphs on OriginPro^33^.

### Ethical approval and consent to participate

We all declare that manuscript reporting studies do not involve any human participants, human data, or human tissue. So, it is not applicable.

Experimental research and field studies on plants (either cultivated or wild), including the collection of plant material, must comply with relevant institutional, national, and international guidelines and legislation.

We confirmed that all methods followed the relevant guidelines/regulations/legislation. The authors have complied with the IUCN Policy Statement on Research Involving Species at Risk of Extinction and the Convention on the Trade in Endangered Species of Wild Fauna and Flora. Seeds were purchased from a certified seed dealer, so no permission is required.

## Results

### Shoot and root length, fresh weight, and dry weight

Under 0 mM NaCl, treatment 2.5% Si-BC2 caused an improvement in shoot length (~ 21 and ~ 7%), root length (~ 15 and ~ 10%), shoot fresh weight (~ 59 and ~ 27%), shoot dry weight (~ 280 and ~ 163%), root fresh weight (~ 50 and ~ 17%,), root dry weight (~ 55 and ~ 29%,), number of leaves (~ 20 and ~ 7%) and number of tillers (~ 266 and ~ 133%) over control and 1% Si-BC1, respectively. It was observed that applying 2.5% Si-BC2 showed an enhancement of ~ 26 and ~ 9% in shoot length, ~ 50 and ~ 30% in root length, ~ 80 and ~ 26% in shoot fresh weight, ~ 304 and ~ 118% in root fresh weight, ~ 283 and ~ 163% in shoot dry weight, ~ 417 and ~ 196% in root dry weight, ~ 14 and ~ 7% number of leaves and ~ 271 and 234% number of tillers compared to control when cultivated in 200 Mm NaCl (Tables 1 and 2).

**Table 1:** Impact of Biochar 1% and 2.5% on the root length, shoot length, root dry weight, shoot dry weight of wheat plant under 200 mM NaCl stress.

Treatments	Shoot length (cm)		Root length (cm)		Shoot FW (g)		Root FW (g)	
	No stress	Salt stress	No stress	Salt stress	No stress	Salt stress	No stress	Salt stress
Control	40.16 ± 0.72bcd	35.5 ± 0.76d	21.73 ± 0.37bc	13.33 ± 1.01e	5.54 ± 0.20c	2.14 ± 0.18e	1.75 ± 0.06b	0.23 ± 0.12d
1% Si-BC1	42.83 ± 0.72bc	38.66 ± 0.88 cd	23.9 ± 0.45ab	17.33 ± 0.44d	7.02 ± 0.24b	2.69 ± 0.11de	2.05 ± 0.04b	0.51 ± .02cd
2.5% Si-BC2	48.66 ± 2.02a	44.66 ± 0.33ab	25 ± 0.28a	20 ± 0.57 cd	8.79 ± 0.57a	3.86 ± 0.28d	2.63 ± 0.13a	0.95 ± 0.13c

Data present the mean ± standard deviation of three replicates.

**Table 2:** Impact of Biochar 1% and 2.5% on the shoot dry weight, root dry weigh, numbers of leaves and number of tillers of wheat plant under 200 mM NaCl stress.

Treatments	Shoot DW (g)		Root DW (g)		Number of leaves		Number of tillers	
	No stress	Salt stress	No stress	Salt stress	No stress	Salt stress	No stress	Salt stress
Control	0.98 ± 0.01b	0.89 ± 0.09b	0.55 ± 0.00b	0.08 ± 0.01d	5 ± 0ab	4.66 ± 0.33b	1 ± 0b	0.36 ± 0.18b
1% Si-BC1	1.60 ± 0.17b	1.46 ± 0.23b	0.71 ± 0.03a	0.16 ± 0.01d	5.33 ± 0.33ab	5 ± 0ab	1.33 ± 0.33b	0.86 ± 0.08b
2.5% Si-BC2	2.75 ± 0.24a	2.54 ± 0.27a	0.85 ± 0.01a	0.36 ± 0.06c	6 ± 0a	5.33 ± 0.33ab	2.66 ± 0.33a	0.99 ± 0.00b

Data present the mean ± standard deviation of three replicates.

### Spikelets per plant, spike length, spike fresh weight, and spike dry weight

In the case of the number of spikelets per plant, spike length, spike fresh weight, and spike dry weight, an improvement of ~ 5, ~ 12, ~ 47, and ~ 35% was observed, respectively, where 2.5% Si-BC2 was applied over control under 0 mM NaCl. Compared to 1% Si-BC1, applying 2.5% Si-BC2 showed ~ 23, ~ 4, ~ 29, and ~ 22% increases in the number of spikelets per plant, spike length, spike fresh weight, and spike dry weight, respectively, at 0 mM NaCl. Furthermore, treatment 2.5% Si-BC2 caused an improvement of ~ 50 and ~ 6% in the number of spikelets per plant, ~ 21 and ~ 13% in spike length, ~ 146 and ~ 70% in spike fresh weight, and ~ 103 and ~ 37% spike dry weight over control and 1% Si-BC1 under 200 mM NaCl (Table 3).

**Table 3:** Impact of Biochar 1% and 2.5% on the Number of spikelet’s, Spike length (cm), spiked (g) and spike fresh weight (g)of wheat plant under 200 mM NaCl stress.

Treatments	Number of spikelets		Spike length (cm)		Spike dry weight (g)		Spike fresh weight (g)	
	No stress	Salt stress	No stress	Salt stress	No stress	Salt stress	No stress	Salt stress
Control	16.33 ± 0.33abc	12.83 ± 0.92d	16.33 ± 0.33abc	12.83 ± 0.92d	0.56 ± 0.02b	0.18 ± 0.01d	1.56 ± 0.13b	0.52 ± 0.07d
1% Si-BC1	17 ± 0ab	14.5 ± 0.28 cd	17 ± 0ab	14.5 ± 0.28 cd	0.72 ± 0.04a	0.30 ± 0.04 cd	1.9 ± 0.00ab	0.72 ± 0.02 cd
2.5% Si-BC2	18.33 ± 0.33a	15.5 ± 0.28bc	18.33 ± 0.33a	15.5 ± 0.28bc	0.83 ± 0.02a	0.44 ± 0.03bc	2.11 ± 0.11a	1.06 ± 0.10c

Data present the mean ± standard deviation of three replicates.

### Chlorophyll contents

**Figure 1:**
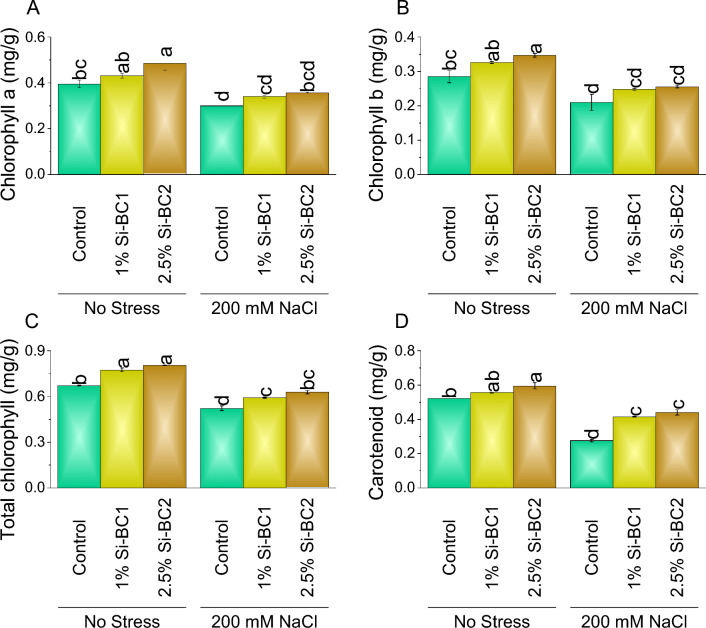
Impact of SiNP-based Biochar different application rates (1% and 2.5%) on chlorophyll a (**A**), chlorophyll b (**B**), total chlorophyll (**C**) and carotenoid (**D**) in wheat leaves under no stress and 200 mM NaCl stress. Data present the mean ± standard deviation of three replicates. Different letters on bars indicate significant differences (*p* ≤ 0.05) compared by Tukey’s Test.

Treatment 1% Si-BC1 showed ~ 9%, ~ 14%, ~ 15%, and ~ 6%, while 2.5% Si-BC2 resulted ~ 23%, ~ 22%, ~ 20%, and ~ 14% enhancement compared to control in chlorophyll a (Fig. 1A), chlorophyll b (Fig. 1B), total chlorophyll (Fig. 1C), and carotenoid (Fig. 1D) respectively under 0 mM NaCl. A significant enhancement was observed with 200 Mm NaCl in chlorophyll a, chlorophyll b, total chlorophyll, and carotenoid where 1% Si-BC1 ~ 13%, ~ 18%, ~ 13%, and ~ 51% and 2.5% Si-BC2 ~ 18%, ~ 22%, ~ 20%, and ~ 60% were applied over control respectively.

### Chlorophyll fluorescence

**Figure 2:**
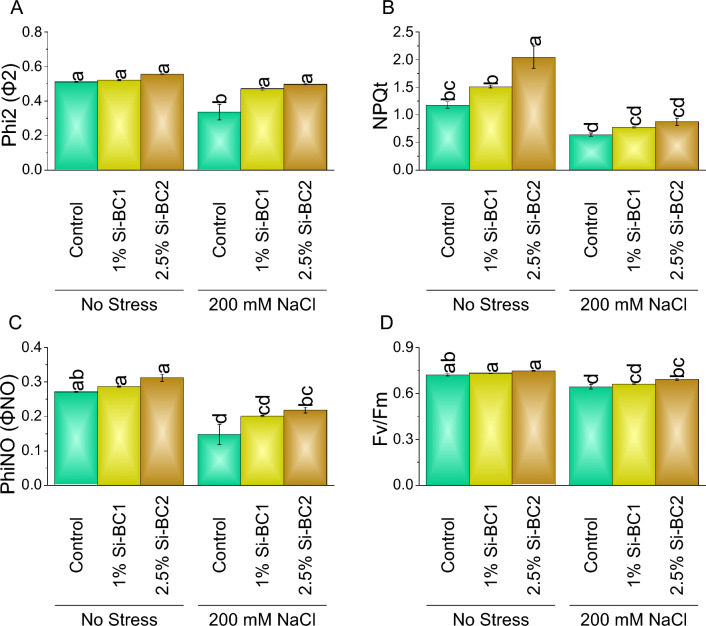
Impact of SiNP-based Biochar different application rates (1% and 2.5%) on Phi-2 (**A**), NPQt (**B**), Fv/Fm (**C**), and PhiNO (**D**) of wheat plant under no stress and 200 mM NaCl stress. Data presents the mean ± standard deviation of three replicates. Different letters on bars indicate significant differences (*p* ≤ 0.05) compared by Tukey’s Test.

1% Si-BC1 showed ~ 2%, ~ 28%, ~ 1%, and ~ 6%, while 2.5% Si-BC2 resulted in ~ 9%, ~ 74%, ~ 4%, and ~ 15% enhancement compared to control in Phi-2 (Fig. 2A), NPQt (Fig. 2B), Fv/Fm, and PhiNO (Fig. 2C) respectively under 0 mM NaCl. A significant enhancement was observed with 200 Mm NaCl in Phi-2, NPQt, Fv/Fm (Fig. 2D), and PhiNO where 1% Si-BC1 ~ 40%, ~ 21%, ~ 3%, and ~ 36% and 2.5% Si-BC2 ~ 48%, ~ 36%, ~ 8%, and ~ 48% were applied over control respectively.

### Antioxidants, electrolyte leakage, and relative water content

**Figure 3:**
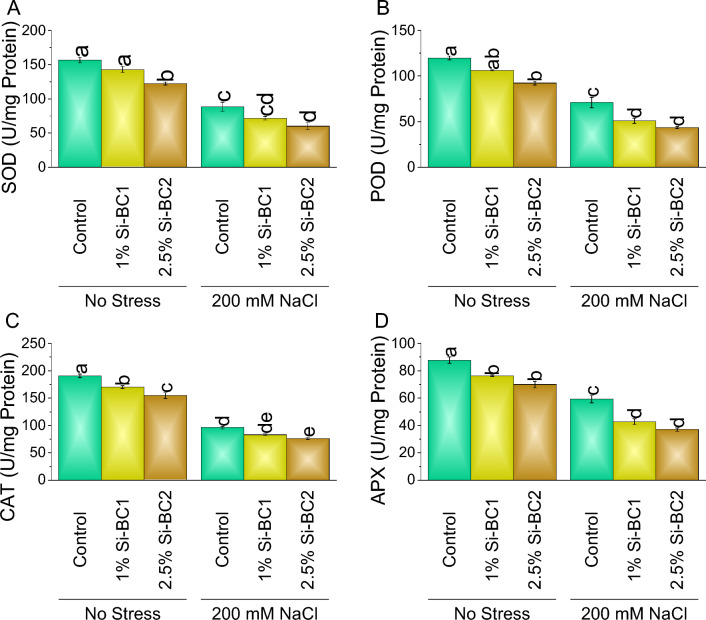
Impact of SiNP-based Biochar different application rates (1% and 2.5%) on SOD (**A**), POD (**B**), CAT (**C**), and APX (**D**) in wheat under no stress and 200 mM NaCl stress. Data presents the mean ± standard deviation of three replicates. Different letters on bars indicate significant differences (*p* ≤ 0.05) compared by Tukey’s Test.

**Figure 4:**
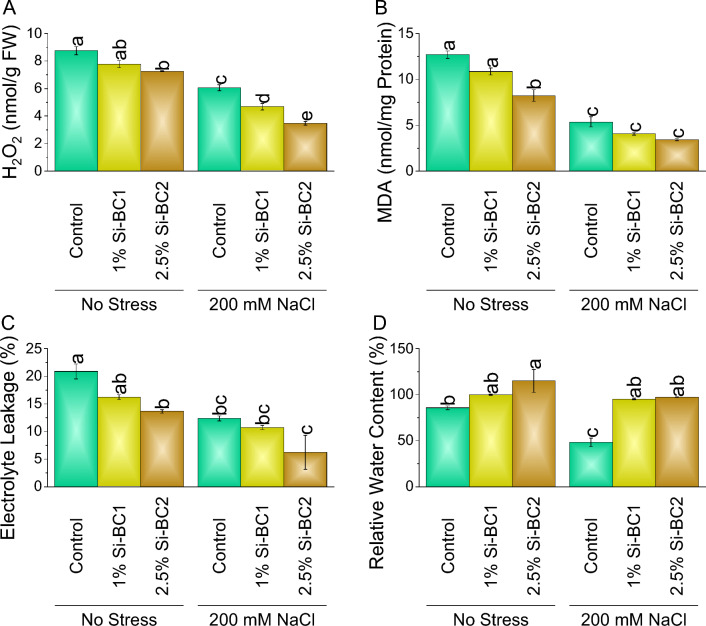
Impact of SiNP-based Biochar different application rates (1% and 2.5%) on H_2_O_2_ (**A**), MDA (**B**), electrolyte leakage (**C**), relative water contents (**D**) in wheat plant under no stress and 200 mM NaCl stress. Data presents the mean ± standard deviation of three replicates. Different letters on bars indicate significant differences (*p* ≤ 0.05) compared by Tukey’s Test.

A significant decline was noted in SOD (Fig. 3A), POD (Fig. 3B), CAT (Fig. 3C), and APX (Fig. 3D) when 1% Si-BC1 ~ 9%, ~ 11%, ~ 11%, and ~ 13%, and ~ 20% and 2.5% Si-BC2 ~ 22%, ~ 23%, ~ 19% and ~ 20% were applied compared to control respectively under no 0 mM NaCl. In the case of 200 mM NaCl, SOD, POD, CAT, and APX showed a decrease of ~ 19%, ~ 28%, ~ 14%, and ~ 28% in 1% Si-BC1, while ~ 32%, ~ 38%, ~ 20%, and ~ 38% in 2.5% Si-BC2 over control (Fig. 3). At 0 mM NaCl, 1% Si-BC1 treatment resulted in ~ 11%, ~ 15%, and ~ 22% reduction in H_2_O_2_, MDA, and electrolyte leakage, respectively, than control. Treatment 2.5% Si-BC2 caused ~ 17%, ~ 35%, and ~ 35% decrease over control in H_2_O_2_ (Fig. 4A), MDA (Fig. 4B), and electrolyte leakage (Fig. 4C), respectively. Furthermore, under 200 Mm NaCl 1% Si-BC1 showed ~ 23%, ~ 24%, and ~ 13%, while 2.5% Si-BC2 resulted in ~ 43%, ~ 36%, and ~ 59% decrease in H_2_O_2_, MDA, and electrolyte leakage, respectively compared to control (Fig. 4). Treatment 1% Si-BC1 treatment resulted in ~ 2% enhancement in relative water content (Fig. 4D), respectively, than control, at 0 mM NaCl. Treatment 2.5% Si-BC2 caused ~ 6% increase over control in relative water content, respectively. Furthermore, with 200 mM NaCl, 1% Si-BC1 showed ~ 55%, while 2.5% Si-BC2 acid resulted in ~ 58% increase in relative water content, respectively, compared to the control.

### Root, shoot and soil macronutrients

**Figure 5:**
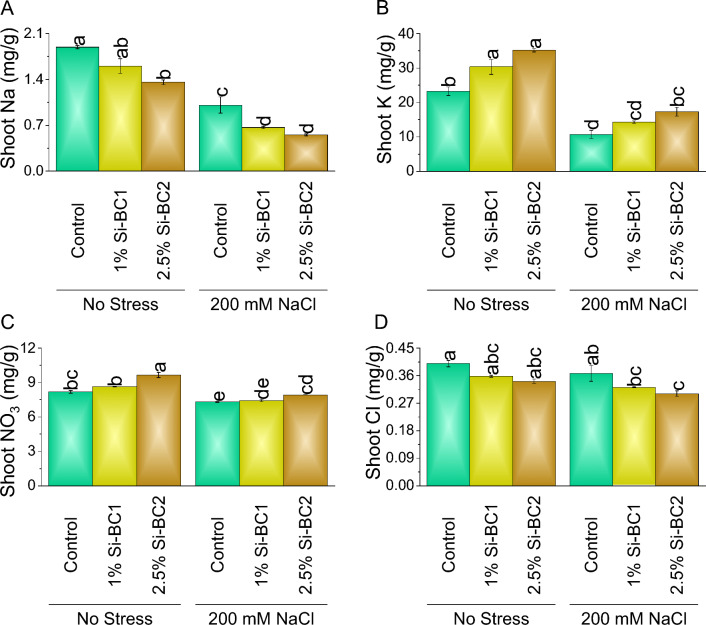
Impact of SiNP-based Biochar different application rates (1% and 2.5%) on shoot Na (**A**), K (**B**), NO_3_ (**C**) and Cl (**D**) concentration of wheat under no stress and 200 mM NaCl stress. Data present the mean ± standard deviation of three replicates. Different letters on bars indicate significant differences (*p* ≤ 0.05) compared by Tukey’s Test.

Si-BC (1%) showed ~ 15% and ~ 10%, while 2.5% Si-BC2 resulted in ~ 28% and ~ 15% reduction compared to control in shoot Na (Fig. 5A) and Cl, respectively, under 0 mM NaCl. While in the case of shoot K (Fig. 5B) and NO_3_ (Fig. 5C), 1% Si-BC1 showed ~ 31% and ~ 6%, while 2.5% Si-BC2 resulted in ~ 51% and ~ 18% enhancement compared to the control, respectively under 0 mM NaCl. A significant increase was observed in 200 Mm NaCl in shoot K and NO_3_, where 1% Si-BC1 ~ 33% and ~ 1%, while 2.5% Si-BC2 ~ 61% and ~ 9% were applied over control, respectively. A significant decrease was observed of 200 Mm NaCl in shoot Na and Cl (Fig. 5D) where 1% Si-BC1 ~ 34%, and ~ 33%, while 2.5% Si-BC2, ~ 45%, and ~ 18% were applied over control.

**Figure 6:**
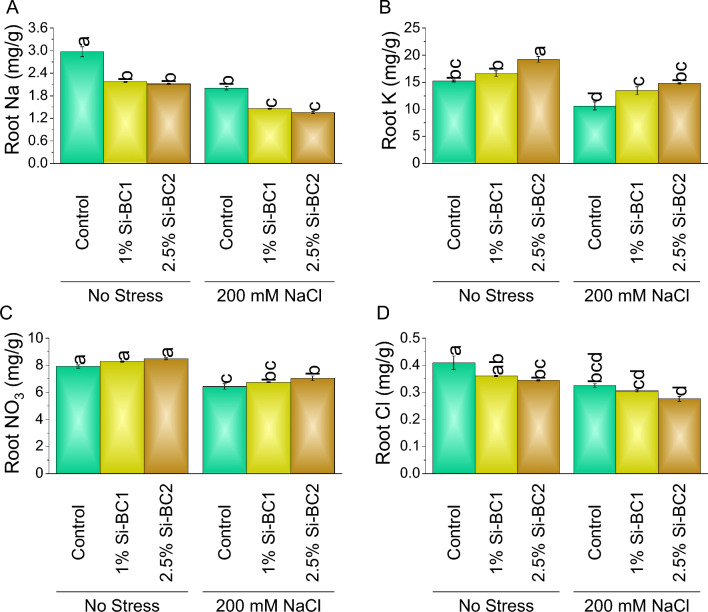
Impact of SiNP-based Biochar different application rates (1% and 2.5%) on root Na (**A**), K (**B**), NO_3_ (**C**) and Cl (**D**) concentration of wheat under no stress and 200 mM NaCl stress. Data present the mean ± standard deviation of three replicates. Different letters on bars indicate significant differences (*p* ≤ 0.05) compared by Tukey’s Test.

Under 0 mM NaCl, 1% Si-BC1 showed ~ 27% and ~ 12%, while 2.5% Si-BC2 resulted in ~ 29%, and ~ 16% reduction compared to control in root Na and Cl, respectively. A significant decrease was observed in 200 Mm NaCl in root Na and Cl where 1% Si-BC1 ~ 27% and ~ 6%, while 2.5% Si-BC2 ~ 33% and ~ 15% were applied over control, respectively (Fig. 6). In the condition of 0 mM NaCl, 1% Si-BC1 showed ~ 5% and ~ 9%, while 2.5% Si-BC2 resulted in ~ 7% and ~ 26% enhancement compared to control in root K and NO_3,_ respectively, 0 mM NaCl. A significant increase was observed in 200 Mm NaCl in root K and NO_3_ where 1% Si-BC1 ~ 5%, and ~ 27%, while 2.5% Si-BC2 ~ 9%, and ~ 39% were applied over control, respectively (Fig. 6A–D).

**Figure 7:**
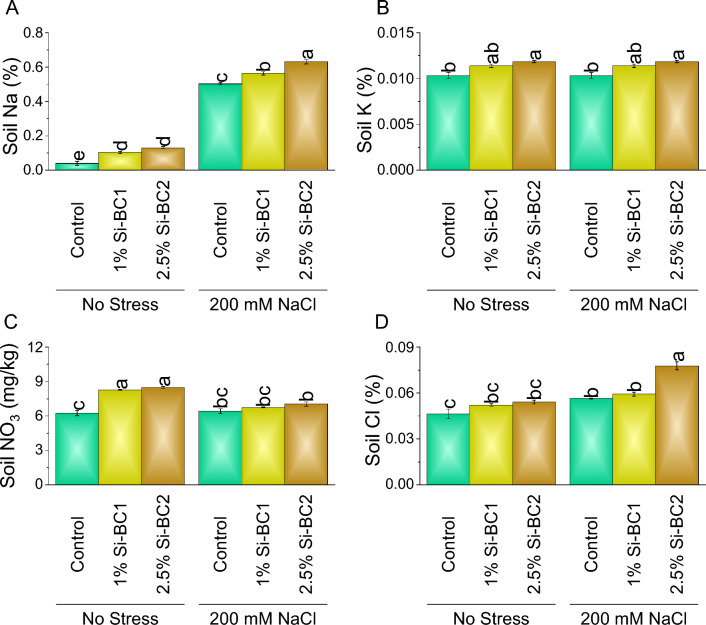
Impact of SiNP-based Biochar different application rates (1% and 2.5%) on soil Na (**A**), K (**B**), NO_3_ (**C**) and Cl (**D**) content under no stress and 200 mM NaCl stress. Data present the mean ± standard deviation of three replicates. Different letters on bars indicate significant differences (*p* ≤ 0.05) compared by Tukey’s Test.

At 0 mM NaCl, 1% Si-BC1 showed ~ 159%, ~ 10%, ~ 32%, and ~ 12%, while 2.5% Si-BC2 resulted in ~ 227%, ~ 15%, ~ 36%, and ~ 17% enhancement compared to control in soil Na, K, NO_3_, and Cl respectively. A significant enhancement was observed in 200 Mm NaCl in soil Na, K, NO_3_, and Cl where 1% Si-BC1 ~ 12%, ~ 10%, ~ 5%, and ~ 5%, while 2.5% Si-BC2 ~ 26%, ~ 14%, ~ 9%, and ~ 38% were applied over control respectively (Fig. 7A–D).

### Root, shoot, and spike micronutrients

For root Zn (~ 35 and ~ 10%), Cu (~ 16 and ~ 2%), Fe (~ 3% and ~ 1%,) and Mn (~ 7 and ~ 3%), a significant improvement was observed where 2.5% Si-BC2 was applied compared to control and 1% Si-BC1 at 0 mM NaCl. At 200 mM NaCl, 2.5% Si-BC2 resulted in an improvement of ~ 35 and ~ 10% in root Zn, ~ 45 and ~ 42% in root Cu, ~ 5 and ~ 1% in root Fe and ~ 6 and ~ 3% in root Mn than control and 1% Si-BC1 (Table 4). At 0 mM NaCl, shoot Zn (~ 9 and ~ 2%), Cu (~ 12 and ~ 2%), Fe (~ 43% and ~ 19%,) and Mn (~ 13 and ~ 3%) were enhanced in 2.5% Si-BC2 than control and 1% Si-BC1. The application of 2.5% Si-BC2 showed an enhancement of shoot Zn (~ 18 and ~ 11%), shoot Cu (~ 21 and ~ 11%), shoot Fe (~ 2 and ~ 5%), and shoot Mn (~ 6 and ~ 2%) compared to the control and 1% Si-BC1, respectively under 200 mM (Table 5). Under 0 mM NaCl, treatment 2.5% Si-BC2 caused an improvement in spike Zn (~ 57 and ~ 14%), Cu (~ 36 and ~ 21%), Fe (~ 4 and ~ 3%) and Mn (~ 9 and ~ 4%) over control and 1% Si-BC1, respectively. It was observed that applying 2.5% Si-BC2 showed an enhancement of ~ 23 and ~ 14% in spike Zn, ~ 23 and ~ 14% in spike Cu, ~ 5 and ~ 4% in spike Fe, and ~ 3 and ~ 1% in spike Mn compared to control when cultivated in 200 Mm NaCl (Table 6).

**Table 4:** Impact of Biochar 1% and 2.5% on the root Zn (%), root Cu (%), root Fe (%) and root Mn (%) of wheat plant under 200 mM NaCl stress.

Treatments	Root Zn (%)		Root Cu (%)		Root Fe (%)		Root Mn (%)	
	No stress	Salt stress	No stress	Salt stress	No stress	Salt stress	No stress	Salt stress
Control	1.1 ± 0d	1.6 ± 0.05bc	107.66 ± 0.88d	202 ± 1b	19.16 ± 0.06d	20.16 ± 0.03bc	6.02 ± 0.01f	6.57 ± 0.01c
1% Si-BC1	1.36 ± 0.03cd	1.76 ± 0.03b	152.66 ± 0.88c	206 ± 1b	19.36 ± 0.03d	20.4 ± 0.05b	6.2 ± 0.07e	6.79 ± 0.00b
2.5% Si-BC2	1.43 ± 0.03c	2.16 ± 0.12a	155.66 ± 0.88c	233.66 ± 2.02a	20.03 ± 0.06c	20.66 ± 0.06a	6.36 ± 0.01d	7.01 ± 0.00a

Data present the mean ± standard deviation of three replicates.

**Table 5:** Impact of Biochar 1% and 2.5% on the shoot Zn (%), shoot Cu (%), shoot Fe (%) and shoot Mn (%) of wheat plant under 200 mM NaCl stress.

Treatments	Shoot Zn (%)		Shoot Cu (%)		Shoot Fe (%)		Shoot Mn (%)	
	No stress	Salt stress	No stress	Salt stress	No stress	Salt stress	No stress	Salt stress
Control	41.66 ± 0.66d	50.33 ± 0.33b	3.23 ± 0.03d	4.03 ± 0.03b	179.66 ± 1.20e	204 ± 1.15c	20.13 ± 0.03d	21.6 ± 0.15c
1% Si-BC1	46.33 ± 1.45c	51.33 ± 0.33ab	3.6 ± 0.05c	4.1 ± 0b	184 ± 1.15de	242.66 ± 1.66b	20.5 ± 0.05d	22.13 ± 0.06b
2.5% Si-BC2	49.33 ± 0.33bc	54.66 ± 0.88a	3.9 ± 0bc	4.5 ± 0.15a	188 ± 0.57d	292 ± 1.15a	21.26 ± 0.08c	24.46 ± 0.18a

Data present the mean ± standard deviation of three replicates.

**Table 6:** Impact of Biochar 1% and 2.5% on the spike Zn (%), spike Cu (%), spike Fe (%), spike Mn (%) of wheat plant under 200 mM NaCl stress.

Treatments	Spike Zn (%)		Spike Cu (%)		Spike Fe (%)		Spike Mn (%)	
	No stress	Salt stress	No stress	Salt stress	No stress	Salt stress	No stress	Salt stress
Control	52.66 ± 0.88d	67.33 ± 0.33c	3.33 ± 0.12e	5.1 ± 0.11c	182 ± 1.15d	203.33 ± 1.20b	34.56 ± 0.20c	37.46 ± 0.68b
1% Si-BC1	56.33 ± 1.33d	81.66 ± 0.88b	3.7 ± 0.05de	6.36 ± 0.14b	188.33 ± 0.88c	207 ± 0.57ab	35 ± 0.05c	38.83 ± 0.14b
2.5% Si-BC2	64.33 ± 0.66c	91.33 ± 1.45a	4.1 ± 0.11d	7.26 ± 0.12a	191.66 ± 0.88c	211.66 ± 1.45a	35.7 ± 0.1c	40.8 ± 0.35a

Data present the mean ± standard deviation of three replicates.

### Morphology and structural analyses of biochar

**Figure 8:**
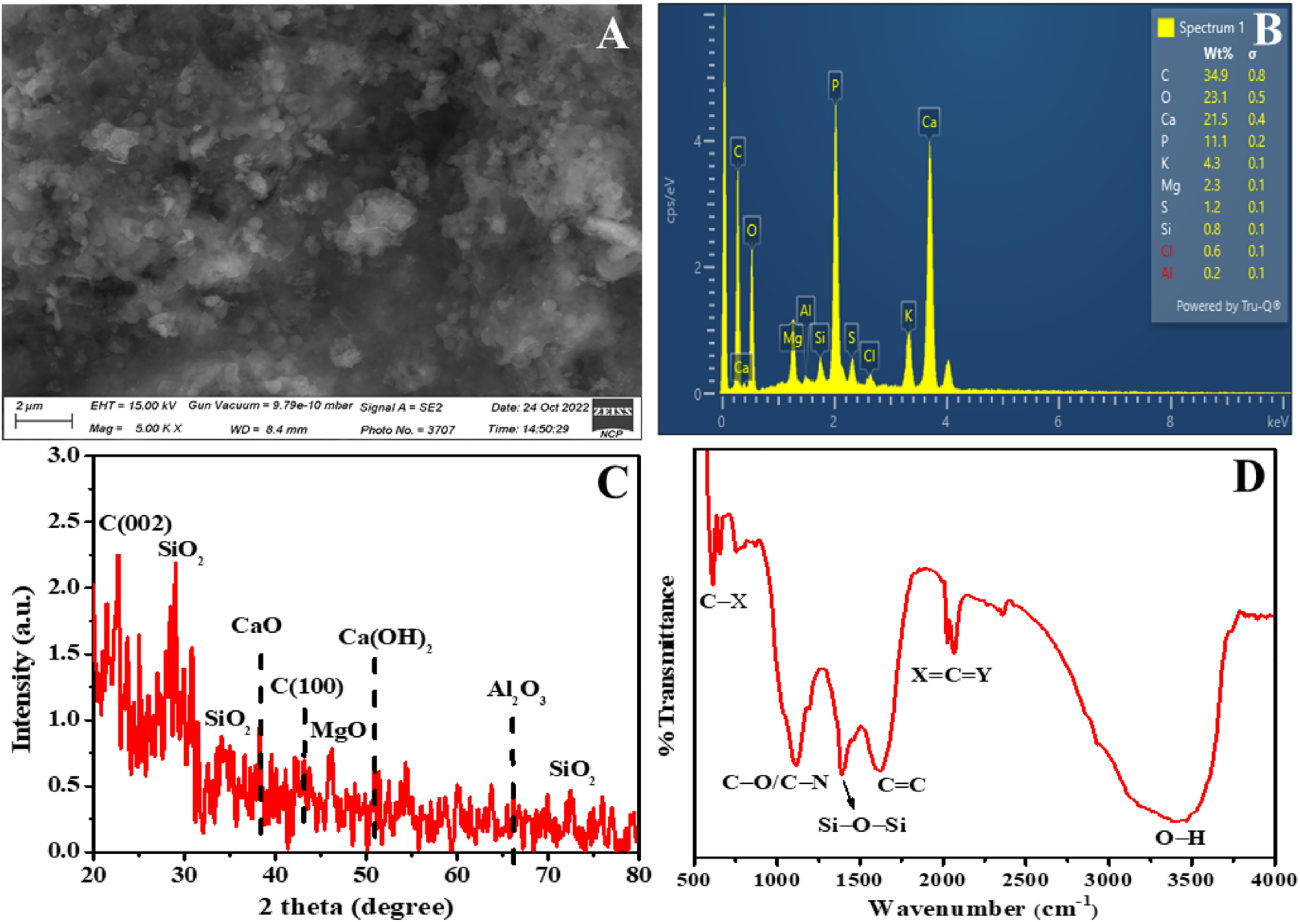
SEM image showing small macropores on the surface of sieved biochar (sBC) with granular features (**A**). EDX map clearly showing traces of various elements in the sample (**B**). The XRD pattern of sBC (**C**). Corresponding FTIR spectrum showing different functional groups present in sBC sample (**D**).

SEM analysis (Fig. 8A) revealed that the sBC sample exhibited a porous and heterogeneous structure with various nanoparticles attached to the biochar surface. The EDX analysis (Fig. 8B) confirmed the presence of multivalent metal elements in the sBC sample, possibly in hydrobiotite minerals. XRD analysis (Fig. 8C) identified crystalline phases in the sBC sample. Peaks corresponding to carbon (e.g., graphene) and silica (quartz) were observed, consistent with the EDX results. Peaks associated with calcium compounds, such as calcite (CaCO_3_), were also detected, indicating potential sites for phosphorous adsorption on the biochar surface. Furthermore, diffraction peaks within the 60°-65° range indicated the presence of kaolinite and hydrobiotite minerals. FTIR spectroscopy (Fig. 8D) provided insights into the functional groups present in the sBC sample. Peaks in the 1600–1650 cm^−1^ range were attributed to C–X bonds, while a sharp peak at 1115 cm^−1^ indicated the presence of C–O/C–N conjugates. Peaks near 1380 cm^−1^ and above 750 cm^−1^ suggested Si–O–Si vibrations and Si–O–Si asymmetric bending vibrations, respectively. A broad vibration centered at about 1615 cm^−1^ indicated C=C stretching due to conjugated carbon, while vibrations in the 2020–2070 cm^−1^ range represented X=C=Y bonding. Vibrations between 3200 and 3400 cm^−1^ indicated O–H bonding in the sBC sample.

## Discussion

Salinity is one of the major abiotic environmental factors that adversely affect crop productivity^34^. Salinity stress dramatically decreased the root and stem dry matter compared to the control treatment because of the direct impacts of ion toxicity or the indirect effects of salty ions that cause soil/plant osmotic imbalance. This judgment agrees with^35^. As a result of the salt effect on the plasma membrane's electrical potential, which decreased both ion and water absorption, creating water stress^36^, when wheat plants exposed to saline conditions showed lower RWC and MSI values. The findings from the present study revealed that saline soil significantly hindered plant growth and reduced wheat's relative water content (RWC) in the absence of Si-BC application. Consistent with these results, numerous other studies have also observed that salinity treatment further impairs plant growth. This could be attributed to the excessive accumulation of sodium, which disrupts water balance, restricts photosynthesis, and damages cell membranes^2,4,5,37^.

The plant was grown in Si-BC amended soil with NaCl treatment, which showed decreased salt stress and boosted plant height, leaf count, and dried fresh weight of the root and shoots. When wheat plants were exposed to 200 mM NaCl salt stress, the Si-BC applied at 1% and 2.5% positively affected growth traits, chlorophyll, leaf fluorescence, and nutrient concentration in above and below-ground (shoot and root) parts of the plant. Previous studies have also revealed the efficacy of biochar in reducing the salinity effects on wheat, sorghum, and maize crops^38–40^. The current study's findings showed that Si-BC increased wheat plant growth and biomass under salt stress by reducing the negative effects of salt stress. Incorporating doped biochar (SBC) significantly enhanced both the plant growth and grain yield of quinoa compared to undoped biochar. This outcome aligns with observations made by researchers in previous studies^41^, who demonstrated that biochar supplemented with silicon (Si) outperformed plain biochar in addressing salt stress. The increased growth observed in response to silicon under salt stress can be attributed to regulating antioxidant enzymes, enhanced nutrient uptake, and modulation of soil pH. Physiological indicators, including chlorophyll content and fluorescence parameters, decreased when plants were subjected to salinity stress. However, adding Si-BC mitigated these declines and improved these physiological attributes. These findings are consistent with earlier research demonstrating that biochar supplementation enhances these traits across various plant species facing salt stress^37^. Our results demonstrated that applying Si-BC caused levels of soluble sugar to increase in salt stress. Sugar plays a role in oxidative stress to eliminate ROS and is a vital component of membranes^42^. Their increased abundance during stressful settings is the breakdown of bigger carbohydrate molecules that keep the cell turgid^43^.

According to recent findings, salinity treatment caused significantly higher levels of H_2_O_2_ and MDA than control plants. Increased levels of H_2_O_2_ and MDA in wheat plants were the signs of oxidative stress^37^. Consistent with these findings, salinity induced oxidative stress and membrane damage in quinoa plants^37^. The inclusion of Si-BC alleviated salinity stress in the plants. The levels of H_2_O_2_ and MDA were reduced, leading to improved stability of cell membranes in the presence of Si-BC. The detoxification of reactive oxygen species (ROS) is facilitated by various antioxidant enzymes within plant organelles^2,17,37^. Antioxidant enzymes are overproduced in the current study under salt stress to lower the levels of ROS, which supports the results of^37,44^. It was discovered that SOD activity increased when exposed to salt stress. Surprisingly, the addition of Si-BC under salinity increased antioxidant enzymes. Different studies have also reported the positive role of silicon nanoparticles doped biochar in increasing the antioxidant activities in plants growing on soil contaminated with NaCl salt^2,17,37^.

The introduction of salinity elevated the sodium (Na) concentration in wheat. Na ions tend to be sequestered in the vacuole rather than expelled by roots. This occurs because, under salinity stress, Na enters plant cells through potassium (K) channels.^45^. Salinity also reduced the uptake of K in quinoa tissues^2,37^. The results of this study highlight the positive impact of Si-BC, which mitigates the accumulation of Na and enhances the uptake of K by wheat plants. Consequently, biochar emerges as an effective strategy for mitigating the adverse effects of salinity on plants by reducing the uptake of toxic ions while increasing the absorption of essential plant nutrients^5,37^. Silicon nanoparticle-based biochar proved even more effective in limiting the accumulation of toxic Na ions and promoting the uptake of essential K ions by wheat. It is well established that Si-BC enhances the uptake of nitrate and chloride due to improved soil nutrient status and facilitates root penetration. Moreover, the presence of various compounds such as magnesium (Mg), calcium (Ca), and phosphorus (P) on the surface of Si-BC enhances cation exchange and water-holding capacity of the soil^46,47^. Our findings indicate that under salt stress, micronutrient uptake decreases. In contrast, biochar supplementation significantly benefits soil health and plant growth by providing essential elements such as iron (Fe), zinc (Zn), and manganese (Mn).

## Conclusion

This is the first study of silicon nanoparticle-based biochar for reducing salinity-induced phytotoxicity in wheat. The current study showed that adding Si-BC to salt-affected soil considerably improved its physicochemical properties, enhancing the physiology and overall growth of *T. aestivum* L. This may be ascribed to improved plant growth, increased water retention, improved nutrient supply, and increased stress tolerance. However, the results were visible when 2.5% Si-BC2 was applied under salt stress. Thus, it was shown that abiotic stresses (such as salinity stress) in the environment could be effectively tolerated by applying various rates of Si-BC; additionally, the adsorption efficiency could be doubled by optimizing it with the application of various types of biochar to improve soil fertility and crop yield. The recent study contributes new information about Si-BC ability to promote plant development in saline soils.

### Supplementary Information

Supplementary Information.

## Data Availability

All data generated or analyzed during this study are included in the article.
