# Supplementary material for: Effect of silicon nanoparticle-based biochar on wheat growth, antioxidants and nutrients concentration under salinity stress
Source: Sci Rep. 2024 Mar 16;14:6380. doi: 10.1038/s41598-024-55924-7 (PMC10944501; doi:10.1038/s41598-024-55924-7)
Supplement: Supplementary file 1 — Supplementary Information. [file 41598_2024_55924_MOESM1_ESM.docx]

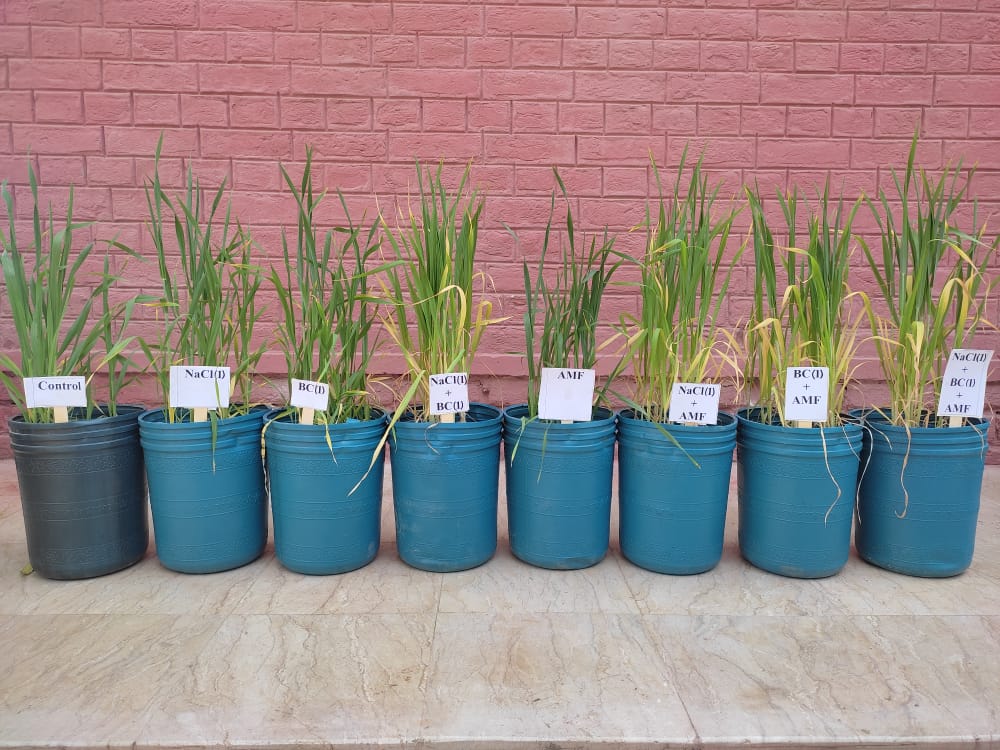


**Figure S1:** Wheat plants during experiment under applied treatments.


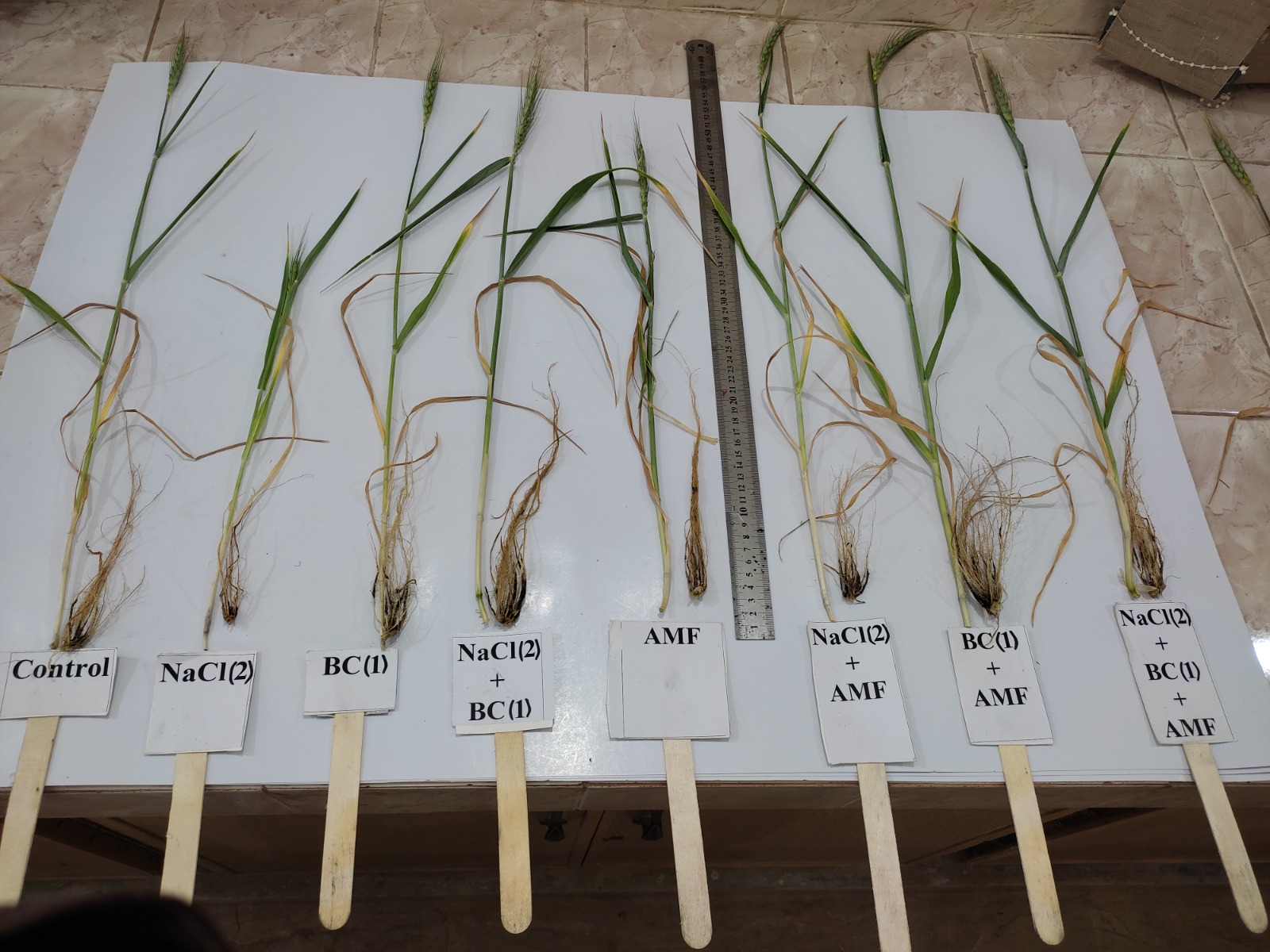


**Figure S2:** Wheat plants after harvesting.
